# The Effects of Hypoxia-Reoxygenation in Mouse Digital Flexor Tendon-Derived Cells

**DOI:** 10.1155/2020/7305392

**Published:** 2020-12-15

**Authors:** Chen Chen, Wei Feng Mao, Ya Fang Wu

**Affiliations:** ^1^Department of Hand Surgery, Affiliated Hospital of Nantong University, Nantong, Jiangsu, China; ^2^Department of Anatomy, Medical School, Nantong University, Nantong, Jiangsu, China

## Abstract

**Objective:**

Ischemia-reperfusion injury refers to the exacerbated and irreversible tissue damage caused by blood flow restoration after a period of ischemia. The hypoxia-reoxygenation (H/R) model in vitro is ideal for studying ischemia-reperfusion injury at the cellular level. We employed this model and investigated the effects of cobalt chloride- (CoCl_2_-) induced H/R in cells derived from mouse digital flexor tendons.

**Materials and Methods:**

Various H/R conditions were simulated via treatment of tendon-derived cells with different concentrations of CoCl_2_ for 24 h, followed by removal of CoCl_2_ to restore a normal oxygen state for up to 96 h. Cell viability was measured using the Cell Counting Kit-8 (CCK-8) assay. Cell growth was determined via observation of cell morphology and proliferation. Oxidative stress markers and mitochondrial activity were detected. The expression levels of hypoxia-inducible factor- (HIF-) 1*α*, vascular endothelial growth factor-A (VEGF-A), collagen I, and collagen III were determined using Western blot (WB), real-time PCR, and immunofluorescence staining. Cellular apoptosis was analyzed via flow cytometry, and the expression of apoptosis-related proteins Bax and bcl-2 was examined using WB.

**Results:**

The cells treated with low concentrations of CoCl_2_ showed significantly increased cell viability after reoxygenation. The increase in cell viability was even more pronounced in cells that had been treated with high concentrations of CoCl_2_. Under H/R conditions, cell morphology and growth were unchanged, while oxidative stress reaction was induced and mitochondrial activity was increased. H/R exerted opposite effects on the expression of HIF-1*α* mRNA and protein. Meanwhile, the expression of VEGF-A was upregulated, whereas collagen type I and type III were significantly downregulated. The level of cellular apoptosis did not show significant changes during H/R, despite the significantly increased Bax protein and reduced bcl-2 protein levels that led to an increase in the Bax/bcl-2 ratio during reoxygenation.

**Conclusions:**

Tendon-derived cells were highly tolerant to the hypoxic environments induced by CoCl_2_. Reoxygenation after hypoxia preconditioning promoted cell viability, especially in cells treated with high concentrations of CoCl_2_. H/R conditions caused oxidative stress responses but did not affect cell growth. The H/R process had a notable impact on collagen production and expression of apoptosis-related proteins by tendon-derived cells, while the level of cellular apoptosis remained unchanged.

## 1. Introduction

Tendon injury is frequently accompanied by a lack of blood supply, exposing the tendon to a hypoxic condition. Oxygen tension, which is influenced by blood supply, is vital to cell survival and proliferation. In vitro studies have shown that low oxygen tension enhances the expansion capacity of newborn pig tenocytes without affecting the cellular phenotype and functions [1], and hypoxic conditions significantly improve the cell proliferation and self-renewal capacity of human tendon stem cells [2, 3]. Other studies, however, have shown that hypoxia inhibits cell proliferation and alters the synthesis of matrix components in synovial tissue [4]. Cobalt chloride- (CoCl_2_-) induced hypoxia has been shown to alter the expression of the bcl-2 family proteins and trigger caspase cascade apoptosis [5, 6]. Collectively, the conflicting results in these studies underscore the complicated effects of hypoxia on cell growth.

Most tissues subjected to oxygen deprivation after injury undergo reperfusion [7–10]. Reperfusion injury, which is manifested by blood flow-deprived and oxygen-starved organs following blow flow restoration and tissue reoxygenation, is a tissue response to stimulate oxidative metabolism [11–15]. Tissues with reperfusion injury share some of the characteristic features of injury responses to oxygen deprivation and subsequent reoxygenation, such as necrosis, apoptosis, impaired microvascular function, and edema. It has been shown that considerable vascular remodeling takes place during tendon healing to enhance perfusion [16, 17]. Stange et al. reported a peak in changes of relative blood volume 7 days after in situ freezing-induced tendinopathy in a rat patellar tendon [18]. A significant uniform hypervascularization occurred during the healing process of chronic Achilles tendinopathy after operative tenolysis in rabbits [17]. The increased vascularization facilitates reoxygenation during tendon repair. Therefore, the process of ischemia/reperfusion after tendon injury, which causes hypoxia-reoxygenation (H/R) changes at the cellular level, presents a major impediment for tendon recovery [19]. However, the sensitivity of tendon-derived cells to hypoxia and the effect of H/R on cell growth and characteristics have been rarely addressed.

In this study, we aimed to establish an in vitro model of cobalt chloride- (CoCl_2_-) induced hypoxia-reoxygenation (H/R) in mouse digital flexor tendon-derived cells and investigate the effects of H/R on these cells [20]. Hypoxic conditions were simulated by treating cultured cells with CoCl_2_, whereas reoxygenation conditions were simulated by replacing the culture media following CoCl_2_ treatment. The principle behind CoCl_2_-induced hypoxia is the replacement of Fe^2+^ by Co^2+^ in heme-based oxygen sensors, which prevents the oxygen sensor from combining with oxygen [21]. Compared with culturing cells in a low-oxygen incubator, the mimic of the hypoxic niche chemically exhibits several advantages, including simple preparation of CoCl_2_, flexible dosage adjustment, treatment suspension at any time, and no impact on oxygen levels for cells that require normal oxygen tension [22]. To have better control over the degree of hypoxia, we decided to employ different CoCl_2_ concentrations. Following H/R, cell viability, cell growth, oxidative stress markers, mitochondrial activity, and expression of HIF-1*α*, vascular endothelial growth factor-A (VEGF-A), collagen I, and collagen III were assessed. Cellular apoptosis and expression of apoptosis-related protein markers Bax and bcl-2 were analyzed.

## 2. Materials and Methods

### 2.1. Experiment Design

We first confirmed the success of the CoCl_2_-induced hypoxia model by examining the expression of HIF-1*α* protein in tendon-derived cells treated with low-to-high concentrations of CoCl_2_ (0.05, 0.1, 0.2, and 0.4 mM), according to previous studies [20, 23, 24]. Next, the cells were divided into the following groups: control (cells cultured in regular medium), H (cells treated with a concentration gradient of CoCl_2_ for 24 h), and R (cells treated with CoCl_2_ for 24 h, followed by culturing in regular culture medium for 24 h, 48 h, 72 h, and 96 h (R24h, R48h, R72h, and R96h)). Cell viability was compared across the control, H, and R groups, as well as among different concentrations of CoCl_2_. Then, we determined an appropriate concentration of CoCl_2_ for the hypoxia model to perform the following experiments: cell growth, oxidative stress markers, mitochondrial activity, mRNA and protein expression of HIF-1*α*, VEGF-A, collagen I, and collagen III, cellular apoptosis, and expression of the apoptosis-related proteins Bax and bcl-2.

### 2.2. Isolation of Mouse Flexor Tendon Cells and H/R Model

The animal procedures were approved by the Administration Committee of Experimental Animals of our university. All animal experiments were carried out in accordance with the Experimental Animal Management Ordinance (National Science and Technology Committee of China) and the Guide for the Care and Use of Laboratory Animals (National Institutes of Health (NIH), Bethesda, MD, USA). Tendon-derived cells were isolated from mouse flexor digitorum profundus (FDP) tendons (C57BL/6, female, 4 weeks old). The mice were sacrificed by cervical dislocation, and the FDP tendons of the index, middle, and ring fingers of the hind paws were harvested. The tendon samples were washed with sterile phosphate-buffered saline (PBS) and then cut into small pieces. The cut samples were digested in a mixture of 3 mg/ml type I collagenase (Gibco, Thermo Fisher Scientific, Grand Island, NY) and 4 mg/ml dispase (Gibco, Thermo Fisher Scientific) for 2 h at 37°C. Undigested tissues and debris were filtered through a 70 *μ*m cell strainer (Merck Millipore, Cork, Ireland). The released cells in the filtrate were centrifuged at 600 g for 10 min and resuspended in low-glucose Dulbecco's modified Eagle's medium (LG-DMEM; Gibco, Thermo Fisher Scientific) supplemented with 10% fetal bovine serum (FBS), 100 U/ml penicillin, and 100 *μ*g/ml streptomycin. The isolated nucleated cells were plated as passage 0 (P0) at a seeding density of 500 cells/cm^2^, as determined in a previous study [25]. The cells were cultured at 37°C with 5% CO_2_. After 2 days, the cells were washed twice with PBS to remove nonadherent cells. After 10 days, the cells at 95% confluence were collected via local trypsin digestion and split 1 : 3 for subsequent passages. The medium was changed twice a week. Cells at P3 were used for all the experiments.

### 2.3. Cell Viability

The cells were seeded in 96-well plates at a density of 5000 cells/well and cultured in DMEM. After 48 h, different concentrations of CoCl_2_ (0.05, 0.1, 0.15, 0.2, 0.25, 0.3, 0.35, and 0.4 mM) were added to the cells for 24 h. Subsequently, the cell media were replaced with regular DMEM, and the cells were cultured for 24 h, 48 h, 72 h, and 96 h. Cell Counting Kit-8 (CCK-8; meilunbio, China) was used to evaluate the viability of cells subjected to H/R. Briefly, fresh medium containing CCK-8 solution was added to the 96-well plates containing cells cultured for different time periods. The cells were then incubated for 1.5 h at 37°C. The absorbance of the supernatants (100 *μ*l) was measured at 450 nm using an automatic microplate reader (Bio-Rad).

### 2.4. Cell Growth

The cells were seeded in a 6-well plate at 5000 cells/cm^2^ and in a 96-well plate at 5000 cells/well. After 48 h, cells were treated with CoCl_2_ (concentration determined by above cell viability measurement) for 24 h, followed by reoxygenation for 24 h, 48 h, 72 h, and 96 h. The morphological changes of cells in the 6-well plate before and after H/R treatment were observed under a phase-contrast microscope (Olympus IX71). The cells in the 96-well plate were harvested using a 0.05% trypsin (Invitrogen) solution, and the cell numbers were counted for growth curve construction before and after H/R treatment.

### 2.5. Oxidative Stress Markers

The intracellular malondialdehyde (MDA) levels were measured using a Lipid Peroxidation MDA Assay Kit (Beyotime, Shanghai, China) following the manufacturer's instructions. First, cells were seeded in 6-well plates (5 × 10^4^ cells/well) and continuously grew until the confluence reached 90%. After H/R treatment, cells were collected and lysed by cell lysis buffer (Beyotime, Shanghai, China) and centrifuged at 10,000 g for 15 min. The supernatants were reacted with the thiobarbituric acid (TBA), and the reaction products were measured spectrophotometrically at 532 nm.

A superoxide dismutase (SOD) assay was performed using the Total Superoxide Dismutase Assay Kit with WST-8 (Beyotime, Shanghai, China). Cells were seeded in 6-well plates (5 × 10^4^ cells/well) and continuously grew until the confluence reached 90%. The cells were treated with H/R conditions_2_. Cells were washed with cold PBS before the addition of SOD buffer solution. Protein concentration was measured using the Enhanced BCA Protein Assay Kit (Beyotime, Shanghai, China). The samples (20 *μ*l) were mixed with the WST-8/enzyme solution (160 *μ*l) and the reaction starting solution (20 *μ*l) and incubated at 37°C for 30 minutes. The absorbance at 450 nm was measured using a microplate reader (Bio-Rad).

### 2.6. MitoTracker Red Staining

Cells were stained with MitoSOX Red Mitochondrial Superoxide Indicator (Yeasen, Shanghai, China) and Hoechst 33342 (Cell Signaling Technologies, Danvers, MA). Cells at 80-90% confluence were treated with H/R conditions_2_. Then, the cells were incubated with MitoSOX Red Mitochondrial Superoxide Indicator for 10 min at 37°C. Then, the cells were washed with PBS and stained with Hoechst 33342 labeling solution (1 : 10000) for 10 min at room temperature. The cells were observed under a fluorescence microscope (Olympus IX71).

### 2.7. Western Blot Analysis

The cells were rinsed twice with cold PBS and then lysed in RIPA buffer (Beyotime, Shanghai, China) containing 1% (*v*/*v*) phenylmethanesulfonyl fluoride (PMSF) (Beyotime, Shanghai, China) and 1% (*v*/*v*) protease and phosphatase inhibitor cocktails (Roche, Mannheim, Germany). The Enhanced BCA Protein Assay Kit (Beyotime, Shanghai, China) was used to measure protein concentrations. The samples were subjected to 10% SDS-polyacrylamide gel electrophoresis and subsequently transferred onto a polyvinylidene difluoride membrane (Millipore Corp., Billerica, Mass.) under 100 volts for 2 h. The membranes were incubated in Tris-buffered saline (TBS: 10 mM Tris-HCl, pH 7.4, 150 mM NaCl) and 0.1% (*v*/*v*) Tween 20 (TBS-Tween) containing 5% (*w*/*v*) dried milk for 1 h and then incubated with the respective primary antibody overnight at 4°C. The primary antibodies against HIF-1*α*, VEGF-A, collagen I, collagen III, Bax, and bcl-2 are described in Table S1. Next, the filters were washed 3 times, 8 min each, with TBS-T and then incubated with a conjugated affinity-purified secondary antibody labeled with IRDye 800v for 1 h at room temperature. Afterwards, the membranes were washed again, and the protein bands were detected with an Odyssey imager (LI-COR, Inc., Lincoln, NE). The intensity of each band was quantified with ImageJ software (NIH, Bethesda, MD, USA). The expression levels of proteins were normalized to *β*-actin.

### 2.8. Real-Time PCR

Total RNA of cells was isolated using TRIzol^®^ Reagent (Ambion, Life Technologies). First-strand cDNA were reverse-transcribed with HiScript II qRT SuperMix plus gDNA wiper (R223-01, Vazyme, China). QRT-PCR was carried out using 1xAceQ qPCR SYBR Green Master Mix (Q111-02, Vazyme, China) according to the manufacturer's instructions. Specific primers were synthesized for the HIF-1*α*, VEGF-A, collagen I, and collagen III mRNAs. GAPDH was used as an internal reference, and the geometric mean of its expression was used for normalization. The sequences of qPCR primers are listed in Table S2. Relative quantification of the target genes was performed in triplicate and analyzed using the 2^−*ΔΔ*Ct^ method.

### 2.9. Immunocytochemistry

The cells were plated at a density of 1 × 10^4^ cells/well on coverglasses in 24-well plates for 2 days. The expression of 4 protein markers, including HIF-1*α*, VEGF-A, collagen I, and collagen III, was measured using immunocytochemistry. Briefly, cells were fixed in 4% paraformaldehyde for 30 min at room temperature. Following washing, the cells were permeabilized with 0.25% Triton X-100 in PBS and blocked with 1% bovine serum albumin (BSA) for 1 h at room temperature. The cells were then incubated with antibodies against HIF-1*α*, VEGF-A, collagen I, and collagen III overnight at 4°C. After 3 washes with PBS, the cells were incubated with Alexa Fluor488 Goat anti-rabbit IgG (H+L) for 2 h at room temperature. Finally, cells were counterstained with Hoechst. The stained cells were then examined under a fluorescence microscope (Olympus IX71).

### 2.10. Flow Cytometry Analysis

Annexin V labeling was used in conjunction with flow cytometry to detect phosphatidylserine on the outer membrane of apoptotic cells. First, the binding buffer was diluted to 1: 4 with deionized water (4 ml binding buffer+12 ml deionized water). Then, the cells were harvested using 0.05% trypsin with no EDTA solution (Invitrogen), washed twice with 4°C prechilled PBS, and resuspended with binding buffer. Next, the cell concentration was adjusted to 1 × 10^6^ cells / ml. The cell suspension (100 *μ*l) was placed in a 5 ml flow tube, and 5 *μ*l annexin V/Alexa Fluor 488 and 10 *μ*l propidium iodide solution were added. After mixing, the mixture was incubated away from light for 15 min at room temperature. After incubation, 400 *μ*l of PBS was added to the reaction tube, and the cells were analyzed using flow cytometry (Attune NxT, Applied Biosystems, CA, USA). For each sample, approximately 10,000 events were counted and analyzed using the FlowJo software (FlowJo, Ashland, OR, USA).

### 2.11. Statistical Analysis

All experiments were repeated at least 3 times for replication. The data were expressed as mean ± SD. The results were analyzed by one-way ANOVA with post hoc Tukey testing or by the *t*-test for pairwise comparisons. All data were analyzed using Prism 5.0b (GraphPad Software, La Jolla, CA, USA). *P* < 0.05 was considered statistically significant.

## 3. Results

### 3.1. CoCl_2_-Induced Hypoxia Elevates HIF-1*α* Expression

**Figure 1:**
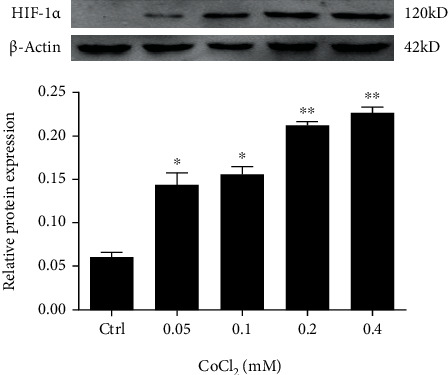
Protein expression of HIF-1*α* after treatment with different concentrations of CoCl_2_ for 24 h. ^∗^*P* < 0.05 vs. control group; ^∗∗^*P* < 0.005 vs. control group.

The protein expression of HIF-1*α* significantly increased in cells treated with CoCl_2_ at concentrations of 0.05 and 0.1 mM (*P* < 0.05) and further increased with higher CoCl_2_ concentrations of 0.2 and 0.4 mM (*P* < 0.005) (Figure 1), which confirmed the success of the CoCl_2_-induced hypoxia model in tendon-derived cells.

### 3.2. H/R Increases Cell Viability

**Figure 2:**
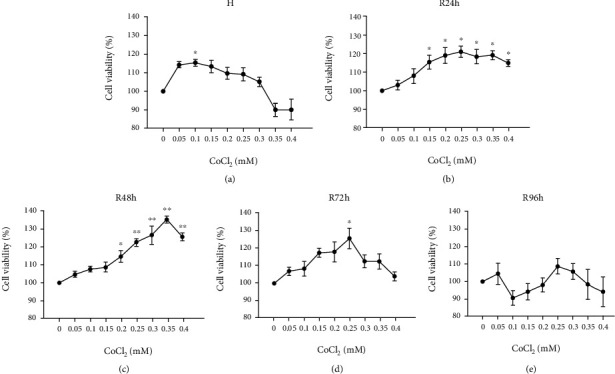
Changes in cell viability of tendon-derived cells under H/R conditions induced by a concentration gradient of CoCl_2_. (a) Cell viability under CoCl_2_-induced hypoxia. (b–e) Cell viability after reoxygenation for 24 h, 48 h, 72 h, and 96 h. ^∗^*P* < 0.05 vs. control group; ^∗∗^*P* < 0.005 vs. control group.

Under hypoxic conditions induced by different concentrations of CoCl_2_, the viability of tendon-derived cells showed an initial increase followed by a gradual decrease with the increased CoCl_2_ concentrations. A significant difference was observed only at 0.1 mM of CoCl_2_ when compared with the control group (*P* < 0.05) (Figure 2(a)). After reoxygenation for 24 h (R24h), cell viability significantly increased in cells treated with 0.15 mM and higher CoCl_2_ concentrations (*P* < 0.05) (Figure 2(b)). At R48h, cell viability significantly increased in cells treated with 0.2–0.4 mM CoCl_2_ (*P* < 0.05 or *P* < 0.005) (Figure 2(c)). At R72h, a significant increase in cell viability was only observed in cells treated with 0.25 mM of CoCl_2_ (*P* < 0.05) (Figure 2(d)). The cell viability of all CoCl_2_-treated cells was similar to that in the control group at R96h (Figure 2(e)).

**Figure 3:**
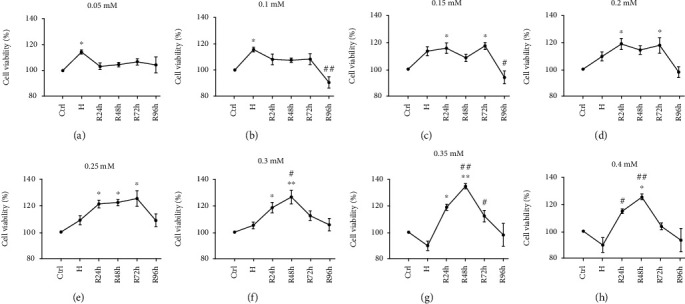
Changes in cell viability of tendon-derived cells under H/R at each concentration of CoCl_2_. (a–h) Cell viability increases along the CoCl_2_ concentration gradient (0.05–0.4 mM). ^∗^*P* < 0.05 vs. control group; ^∗∗^*P* < 0.005 vs. control group; ^#^*P* < 0.05 vs. H group; ^##^*P* < 0.005 vs. H group.

At each concentration, cell viability was compared across the control, hypoxia, and reoxygenation groups. Compared with the control group, the cells treated with low concentrations of CoCl_2_ showed significant increases in cell viability under hypoxia, with a 14% increase at 0.05 mM and 15% at 0.1 mM (*P* < 0.05) (Figures 3(a) and 3(b)). The cells treated with higher concentrations of CoCl_2_ at 0.15, 0.2, 0.25, and 0.3 mM exhibited significantly enhanced cell viability after reoxygenation for 24 h–72 h (*P* < 0.05 or *P* < 0.005) (Figures 3(c)–3(f)). At the 2 highest CoCl_2_ concentrations, cell viability significantly increased at R48h compared with those in the control and H groups (*P* < 0.005) (Figures 3(g) and 3(h)).

### 3.3. H/R Does Not Inhibit Cell Growth

**Figure 4:**
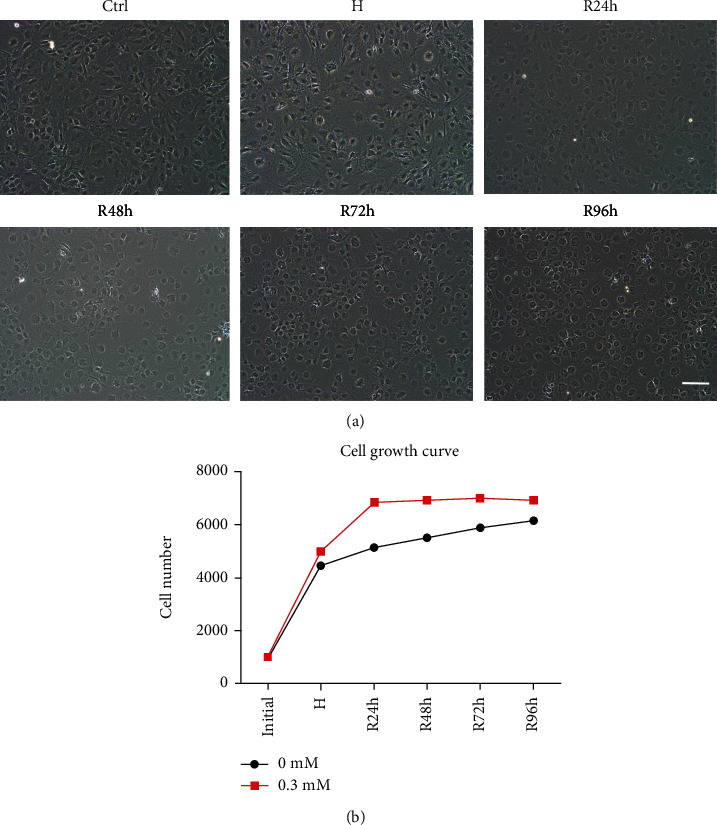
Cell morphology and growth curves of tendon-derived cells when treated with 0.3 mM CoCl_2_. (a) Cell morphology under H/R conditions. Scale bar: 200 *μ*m. (b) Cell growth curves under H/R conditions.

During the experiment, we observed relatively healthy growth of cells treated with 0.3 mM CoCl_2_ after reoxygenation. The cells were shrunk slightly when under hypoxic conditions, but the morphology returned to normal after reoxygenation (Figure 4(a)). Therefore, we selected 0.3 mM CoCl_2_ to simulate hypoxia for subsequent experiments. Cell growth curves revealed a slightly increased cell proliferation under H/R compared with that of the control group (Figure 4(b)).

### 3.4. H/R Mediates Oxidative Stress and Mitochondrial Activity

**Figure 5:**
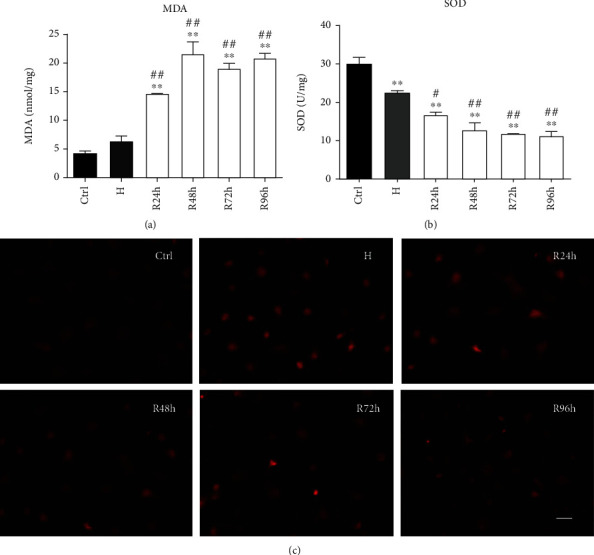
(a) Cellular malondialdehyde (MDA) levels. (b) Superoxide dismutase (SOD) activity. (c) The intensity of mitochondria in different groups. Scale bar: 100 *μ*m. ^∗^*P* < 0.05 vs. control group; ^∗∗^*P* < 0.005 vs. control group; ^#^*P* < 0.05 vs. H group; ^##^*P* < 0.005 vs. H group.

To determine the impact of H/R on oxidative stress, the intracellular levels of MDA and SOD were measured. The MDA levels significantly increased during reoxygenation compared with those in the control and H groups (*P* < 0.005) (Figure 5(a)). The SOD levels significantly decreased under H/R compared with that in the control group (*P* < 0.005) (Figure 5(b)). The MitoTracker Red staining showed that the intensity of bioactive mitochondria was higher in the H and R groups than in the control group (Figure 5(c)).

### 3.5. H/R Enhances Protein Expression of HIF-1*α* and VEGF-A and Decreases Expression of Collagens I and III

**Figure 6:**
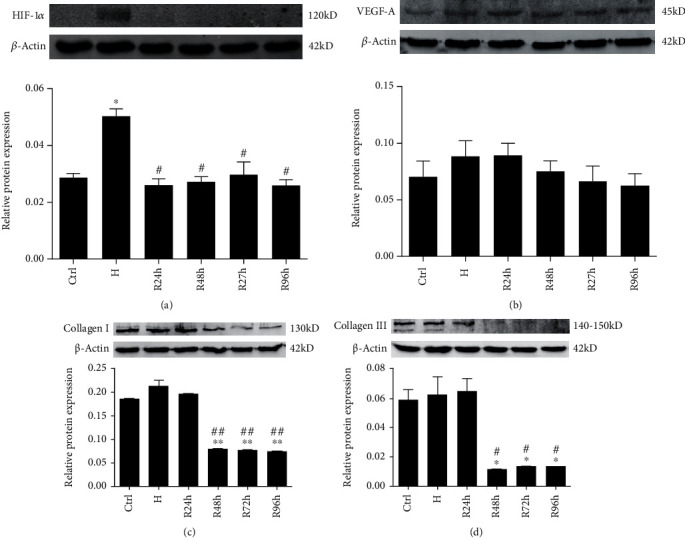
Protein expression of (a) HIF-1*α*, (b) VEGF-A, (c) collagen I, and (d) collagen III when cells were under H/R conditions at 0.3 mM CoCl_2_. ^∗^*P* < 0.05 vs. control group; ^∗∗^*P* < 0.005 vs. control group; ^#^*P* < 0.05 vs. H group; ^##^*P* < 0.005 vs. H group.

The protein expression of HIF-1*α* significantly increased when the cells were under hypoxia and returned to normal levels after reoxygenation (*P* < 0.05) (Figure 6(a)). By comparison, VEGF-A levels showed a similar but not significant increase under hypoxia and at R24h and returned to normal from R48h and after (Figure 6(b)). Both collagen I and collagen III protein levels significantly decreased at R48h, R72h, and R96h compared with those of the control group and H group (*P* < 0.05 or *P* < 0.005) (Figures 6(c) and 6(d)).

### 3.6. H/R Regulates mRNA Expression of HIF-1*α*, VEGF-A, Collagen I, and Collagen III

**Figure 7:**
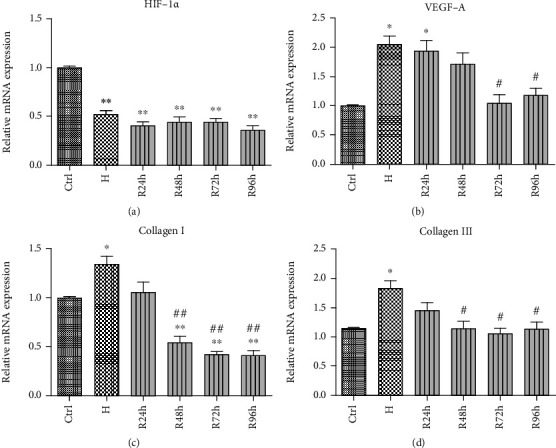
mRNA expression of (a) HIF-1*α*, (b) VEGF-A, (c) collagen I, and (d) collagen III when cells were under H/R conditions induced by 0.3 mM CoCl_2_. ^∗^*P* < 0.05 vs. control group; ^∗∗^*P* < 0.005 vs. control group; ^#^*P* < 0.05 vs. H group; ^##^*P* < 0.005 vs. H group.

Under H/R conditions, the mRNA expression of HIF-1*α* was significantly suppressed (Figure 7(a)). The VEGF-A mRNA level significantly increased when the cells were under hypoxia and at R24h compared with that of the control group (*P* < 0.05) but significantly decreased when the cells were at R72h and R96h compared with that of the H group (*P* < 0.05) (Figure 7(b)). The mRNA expression of collagen I significantly increased under hypoxia, returned to normal levels at R24h, and significantly decreased from R48h to R96h compared with those of the control and H groups (*P* < 0.05 or *P* < 0.005) (Figure 7(c)). The collagen III mRNA levels also significantly increased under hypoxia compared with that of the control group but remained at normal levels with different periods of reoxygenation (*P* < 0.05) (Figure 7(d)).

### 3.7. Immunocytochemical Staining

**Figure 8:**
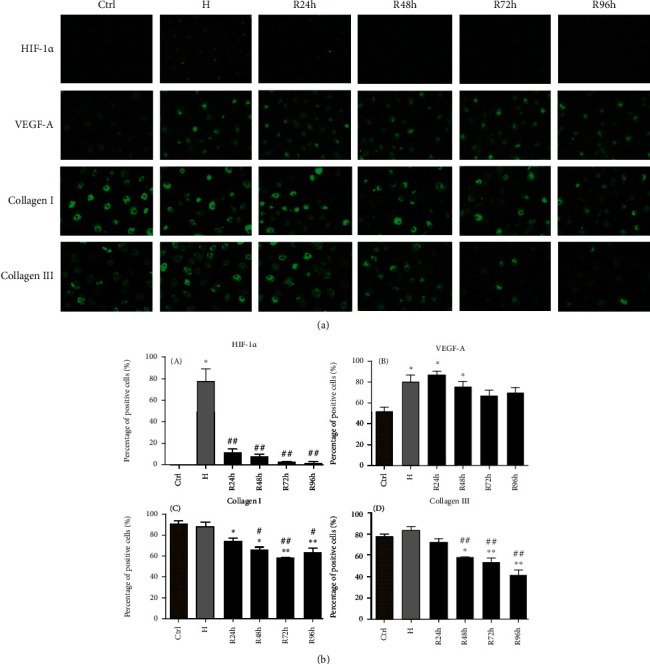
(a) Representative fluorescence micrographs of HIF-1*α*, VEGF-A, collagen I, and collagen III in cells under H/R conditions induced by 0.3 mM CoCl_2_. (b) Percentages of positive cells stained with HIF-1*α* (A), VEGF-A (B), collagen I (C), and collagen III (D). Scale bar: 100 *μ*m. ^∗^*P* < 0.05 vs. control group; ^∗∗^*P* < 0.005 vs. control group; ^#^*P* < 0.05 vs. H group; ^##^*P* < 0.005 vs. H group.

Figure 8(a) shows the representative fluorescence micrographs of HIF-1*α*, VEGF-A, collagen I, and collagen III when the cells were under H/R conditions. We calculated the percentage of positive cells via cell counting Figure 8(b). Compared with the control group, the percentage of HIF-1*α*-positive cells significantly increased under hypoxia (Figure 8(, whereas that of VEGF-A-positive cells was significantly increased under hypoxia, at R24h, and at R48h (*P* < 0.05) (Figure 8(CID="C002" value="B")). The percentages of collagen I- and collagen III-positive cells were significantly lower at R48h, R72h, and R96h compared with those in the control and H groups (*P* < 0.05 or *P* < 0.005) (Figures 8(CID="C004" value="C") and 8(CID="C006" value="D")).

### 3.8. H/R Does Not Affect Cellular Apoptosis but Alters Protein Expression of Bax and bcl-2

**Figure 9:**
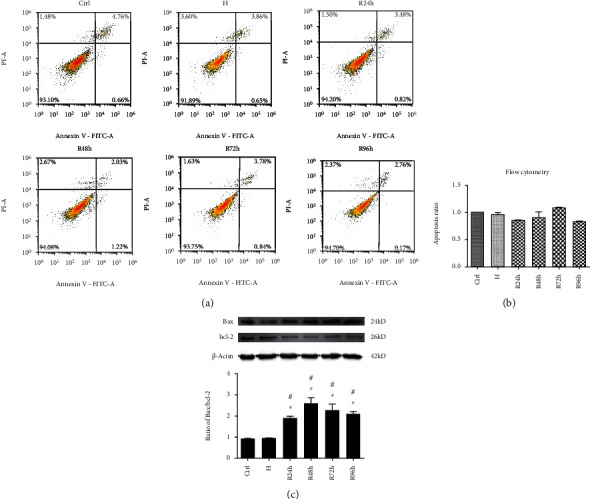
Flow cytometric analysis of cellular apoptosis and ratios of Bax/bcl-2 proteins: (a) representative graphs of flow cytometric assays showing the apoptosis of different groups; (b) the histogram showing the ratio of apoptotic cells; (c) expression ratio of Bax/bcl-2 protein. ^∗^*P* < 0.05 vs. control group; ^∗∗^*P* < 0.005 vs. control group. ^#^*P* < 0.05 vs. H group; ^##^*P* < 0.005 vs. H group.

value="Flo"value="w" cytometry was conducted to investigate the apoptotic levels of cells under H/R conditions. The flow cytometric analysis showed that no significant differences in the number of apoptotic cells were found across the control, hypoxia, and reoxygenation groups (Figures 9(a) and 9(b)). Meanwhile, the ratio of Bax/bcl-2 protein expression was significantly upregulated after reoxygenation compared with those of the control and hypoxia groups (*P* < 0.05), with a peak at R48h (Figure 9(c)).

## 4. Discussion

The current study uncovered the cellular responses of tendon-derived cells to hypoxia and subsequent reoxygenation. Our results showed that different concentrations of CoCl_2_ exerted distinct effects on cell viability. Hypoxia induced by low CoCl_2_ concentrations (0.05 and 0.1 mM) enhanced cell viability, while further increases in CoCl_2_ concentrations (0.15–0.4 mM) led to a gradual decrease in cell viability. With 24 h–72 h of reoxygenation, cell viability increased in cells treated with high concentrations of CoCl_2_. Cell viability returned to the same level as the control group after 96 h of reoxygenation, irrespective of the initial CoCl_2_ concentrations.

Previous studies have demonstrated that different cell lines show diverse tolerance profiles to CoCl_2_-induced H/R. Zhang et al. demonstrated that CoCl_2_ did not promote or attenuate the viability of A498 cells at low concentrations (0.05–0.2 mM), but when the concentration was increased to 0.25 mM, cell activity gradually declined [20]. The study by Shi et al. in a human ovarian carcinoma cell line showed that CoCl_2_-induced (0.15 mM) hypoxia inhibited cell proliferation, which was subsequently recovered with reoxygenation [23]. Tong et al. showed that HepG2 cells treated with different concentrations of CoCl_2_ (0.05, 0.1, 0.15, and 0.2 mM) exhibited significantly repressed cell viability in a concentration-dependent manner [24]. However, our data showed that cell viability was enhanced by CoCl_2_-induced hypoxia, even when the concentration of CoCl_2_ reached 0.3 mM. Furthermore, during the early period of reoxygenation, cell viability further increased, especially in cells treated with high concentrations of CoCl_2_ (0.25–0.4 mM). In addition, H/R had no obvious negative effects on cell growth. These results suggested that tendon-derived cells were highly tolerant to CoCl_2_-induced hypoxia and the reoxygenation.

H/R conditions can cause oxidative stress, which is a major mechanism involved in the pathogenesis of H/R injury [26–29]. Therefore, we examined the levels of two oxidative stress markers, MDA and SOD, and the activity of bioactive mitochondria in cells under H/R. MDA, a byproduct of lipid oxidation, is a biomarker of oxidative stress of cells [30]. SOD is the main antioxidant enzyme in cells that plays important roles in scavenging oxygen free radicals and resisting the damage of oxygen free radicals [31]. Mitochondria, accounting for the majority of oxygen consumption, can help to tune cellular and organismal hypoxia responses [32]. Our results showed that the MDA level increased, the SOD level decreased, and the activity of bioactive mitochondria was enhanced under H/R, indicating that H/R induced notable oxidative stress in the tendon-derived cells. However, the oxidative stress did not affect the cell growth in this study. We speculate that the increased mitochondrial activity may retain the metabolic demand of the cells and induce an adaptive response to oxidative stress [33–36].

HIF-1*α* is a key transcription factor in response to hypoxic stress and is widely expressed in mammals, including humans, under hypoxic conditions [37, 38]. In the present study, the HIF-1*α* protein level increased notably in the cells under hypoxia and then returned to the same level in the control cells immediately after reoxygenation. We noted, however, that the changes in HIF-1*α* mRNA and protein expression were inconsistent. Specifically, HIF-1*α* mRNA expression decreased significantly during hypoxia and reoxygenation, which is inconsistent with previous studies [39, 40]. A possible explanation is that the expression of HIF-1*α* mRNA changes prior to the expression of protein, and the peak of mRNA expression occurred in less than 24 hours. Then, the inhibited HIF-1*α* mRNA expression caused the recovery of the HIF-1*α* protein level in the cells under reoxygenation. These results indicate that tendon-derived cells can regulate the expression of HIF-1*α* rapidly under H/R conditions. It may be beneficial to the adaptation of tendon-derived cells to oxygen deprivation, leading to the high tolerance of this type of cells to H/R.

VEGF-A is an essential growth factor for most tissues in their response to traumatic injuries involving disrupted blood supply. Previous studies demonstrated that the expression of the VEGF gene significantly increased during the early period of tendon healing and that intraoperative delivery of VEGF notably enhanced tendon healing in a chicken model, indicating the importance of VEGF in tendon healing [41, 42]. In our study, we found that VEGF-A was upregulated under hypoxia when HIF-1*α* was activated, and its levels returned to normal during reoxygenation along with the passage of time. Our results were consistent with previous studies investigating the role of the HIF-1*α*/VEGF pathway in hypoxia. Liang et al. showed that hypoxia markedly upregulated VEGF-A and that appropriate vascular response might be essential for normal repair and remodeling. VEGF-A elevation is a rapid and strong response to hypoxic insult typically seen in most tissues [43]. Therefore, hypoxia-promoted expression of VEGF may be a self-protective mechanism after tendon injury.

Collagen is the major substance secreted by tendon cells. It is the main component of tendon ECM, and thus, synthesis of collagen is essential for maintaining tendon structure [44, 45]. A normal tendon comprises 65%–85% of collagen I, which is the most abundant collagen, followed by collagen III [46, 47]. Collagen III is highly upregulated during tendon healing [48]. Collagen III primarily forms an abundant but disorganized collagen matrix in the proliferative phase of tendon healing. Webster et al. showed that when tendon-derived cells were cultured at low oxygen tensions, cellular metabolism was depressed and both total protein and collagen production were reduced [49]. It was hypothesized that the hypoxic environment might not have satisfied the physiological oxygen requirements of the cells, and it may have deleterious effects on collagen production [50]. A study also reported that there was an abrupt reduction of collagens I and III in the early stages of Achilles tendinitis; the tendon subsequently developed substantial impairment and Achilles tendinitis eventually occurred [51]. Our results showed that the expression of collagens I and III was downregulated during reoxygenation. Therefore, the in vitro reoxygenation process may correspond to the in vivo repair process after ischemia. Interestingly, the decline in the expression of collagen appears to contradict the increased cell viability. We speculate that although cell growth is not affected, other cellular functions are inhibited under hypoxic stress.

Apoptosis plays a critical role in the homeostasis of normal tissue [52]. In human rotator cuff tendinopathy, HIF-1*α* accumulation was associated with cellular apoptosis, which provided an early support for the role of hypoxia-induced damage to cell loss via apoptosis [53]. However, Sasabe et al. reported that forced expression of HIF-1*α* suppressed hypoxia-induced apoptosis of human oral squamous cell carcinoma cell lines [54]. Based on flow cytometry analysis in the present study, CoCl_2_-induced H/R had a minimal effect on the cellular apoptosis. However, the protein expression of Bax was upregulated while that of bcl-2 was downregulated, resulting in a significant increase in the Bax/bcl-2 ratio during reoxygenation. The Bax/bcl-2 ratio reached its peak at 48 h after reoxygenation and began to decrease thereafter. This suggested that although the apoptotic pathway was activated, the cells did not actually undergo apoptosis within the observed period. We speculate that tendon-derived cells are highly resistant to H/R-induced apoptosis, and their inherent cell characteristics may prevent or even reverse the occurrence of apoptosis. Another possibility is that the initiation of the apoptotic mechanism precedes the occurrence of apoptosis. To this regard, extending the observation time may allow us to obtain a more complete picture of apoptotic events after H/R.

## 5. Conclusions

Collectively, the present study successfully established a CoCl_2_-induced H/R model in tendon-derived cells, which provided a framework for future studies to understand the tendon-specific features of this widely observed stress response mechanism. The data in this study showed that hypoxia followed by reoxygenation for a certain period of time promoted cell viability in a concentration-dependent manner. Tendon-derived cells exhibited considerable tolerance to hypoxia. H/R caused oxidative stress responses but did not affect cell growth. H/R altered the expression of HIF-1*α*, VEGF-A, collagen I, and collagen III. Cellular apoptosis was not affected by H/R, but the Bax/bcl-2 ratio increased during reoxygenation.

## Data Availability

All data generated or analyzed during this study are included in this published article.

## Supplementary Materials

Supplementary Materials Table S1: antibodies used in the Western blot assay and immunocytochemical staining. Table S2: sequences of qPCR primers.
