# Supplementary material for: The Effects of Hypoxia-Reoxygenation in Mouse Digital Flexor Tendon-Derived Cells
Source: Oxid Med Cell Longev. 2020 Dec 15;2020:7305392. doi: 10.1155/2020/7305392 (PMC7787768; doi:10.1155/2020/7305392)
Supplement: Supplementary Materials — Table S1: antibodies used in the Western blot assay and immunocytochemical staining. Table S2: sequences of qPCR primers. [file 7305392.f1.docx]

**Table S1 Antibodies used in Western blot assay and immunocytochemical staining**

| **Antibody** | **Catalog** | **Host** | **Dilution** |
| --- | --- | --- | --- |
| HIF-1α | Cell signaling #36169 | Rabbit | WB:1000 ICC:200 |
| VEGF | Proteintech 26157-AP | Rabbit | WB:1000 ICC:200 |
| Collagen1 | Abcam ab34710 | Rabbit | WB:1000 ICC:200 |
| Collagen3 | Proteintech 22734-1-AP | Rabbit | WB:1000 ICC:500 |
| Bax | Proteintech 50599-2-lg | Rabbit | WB:1000 |
| Bcl-2 | Proteintech 60178-1-lg | Mouse | WB:1000 |
| β-actin | Abcam ab6276 | Mouse | WB:5000 |
| Alexa Fluor488 Goat anti-Rabbit IgG (H+L) | FcMACS fMS-RBaf48801 | Goat | ICC:200 |

**Table S2 The sequences of qPCR primers**

| **Primer** | **Forward primer** | **Reverse primer** |
| --- | --- | --- |
| HIF-1α | TCTCGGCGAAGCAAAGAGTC | AGCCATCTAGGGCTTTCAGATAA |
| Vegfa | CTTTTCGTCCAACTTCTGGGCTCTT | CCTTCTCTTCCTCCCCTCTCTTCTC |
| Collagen1 | CTGGCGGTTCAGGTCCAAT | TTCCAGGCAATCCACGAGC |
| Collagen3 | CCTGGCTCAAATGGCTCAC | GACCTCGTGTTCCGGGTAT |
| GAPDH | GGTCGGTGTGAACGGATTTG | TGAGTGGAGTCATACTGGAACAT |
